# Supplementary material for: Exploration of the close chemical space of tryptophan and tyrosine reveals importance of hydrophobicity in CW-photo-CIDNP performances
Source: Magn Reson (Gott). 2021 May 12;2(1):321–9. doi: 10.5194/mr-2-321-2021 (PMC12314778; doi:10.5194/mr-2-321-2021)
Supplement: The supplement related to this article is available online at: https://doi.org/10.5194/mr-2-321-2021-supplement. [file mr-2-321-2021-supplement.pdf]

Supplement of Magn. Reson., 2, 321–329, 2021
https://doi.org/10.5194/mr-2-321-2021-supplement
© Author(s) 2021. CC BY 4.0 License.

Supplement of

Exploration of the close chemical space of tryptophan and tyrosine reveals importance of hydrophobicity in CW-photo-CIDNP performances

Felix Torres et al.

Correspondence to: Roland Riek (roland.riek@phys.chem.ethz.ch)

The copyright of individual parts of the supplement might differ from the article licence.

Table S1: Principal photo-CIDNP active known molecules. The non-exhaustive list of used photosensitizers is reported as flavin mononucleotide (FMN), bipyridyl (BIPY), fluorescein (FLUO), Atto Thio 12 (AT12), 3,3',4,4'-tetracarboxy-benzophenone (TCBP).

Molecule	Dye
Tryptophan	FMN[a], BIPY, FLUO[f], AT12[g], TCBP[e]
NAc-tryptophan	FMN[a], TCBP[e]
1-methyl-tryptophan	FMN[a]
Indole	FMN[a]
NAc-serotonin	FMN[a]
Methoxy-tryptamine	FMN[a]
Tyrosine	FMN[a], BIPY, FLUO[f], AT12[g], TCBP[e]
3-NO_2-tyrosine	FMN[a]
3-F-tyrosine	FMN[a]
3-amino-tyrosine	FMN[a]
NAc-tyrosine	FMN[a], TCBP[e]
Histidine	FMN[c], TCBP[e]
NAc-histidine	FMN[a], BIPY[d], TCBP[e]
1-methyl-histidine	FMN[a]
Methionine	FMN[a]
Adenine	FMN[b,c]
Guanine	FMN[b,c]
3-methyl-cytosine	FMN[b]
5-methyl-cytosine	FMN[b]
Thymine	FMN[b,c]
Porphyrin	1,4 benzoquinone[c]
polyphenol	FMN[c]

a) (Stob and Kaptein, 1989); b) (Kaptein et al., 1979) c) (Hore and Broadhurst, 1993) d) (Tsentalovich et al., 2000) e) (Saprygina et al., 2014) f) (Okuno and Cavagnero, 2016) g) (Sobol et al., 2019)

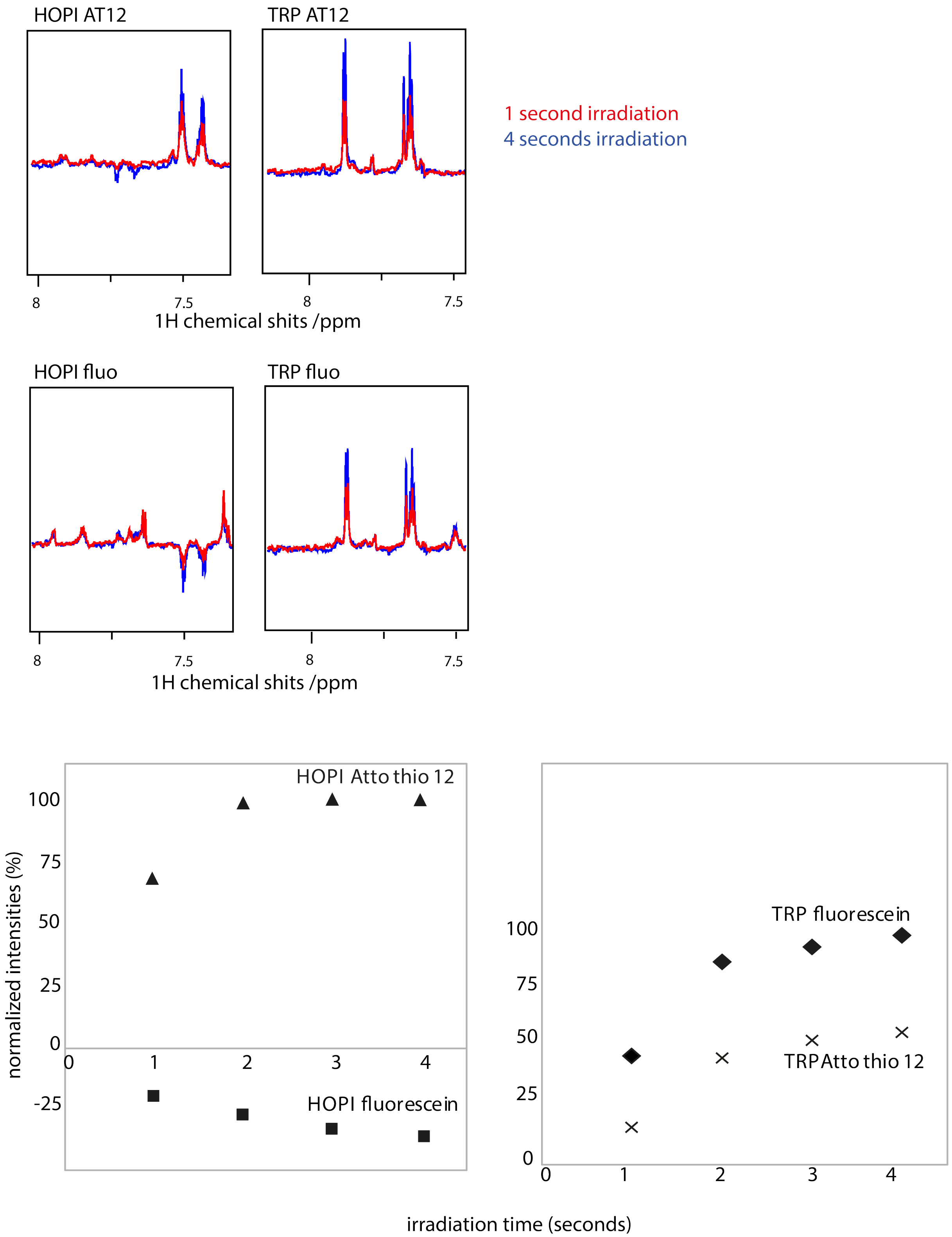

Figure S1: Polarization is dependent on the irradiation time in CW-photo-CIDNP experiments. Top: Photo-CIDNP spectra of HOPI and TRP in the presence of AT12 or fluorescein at 1 and 4 second irradiation time. The respective anomalous line intensity build up plots measured at 600 MHz ^{1}H frequency are depicted in the bottom image. The spectra were measured at 0.05 mM molecule concentration. As demonstrated the polarization is a function of the irradiation time.

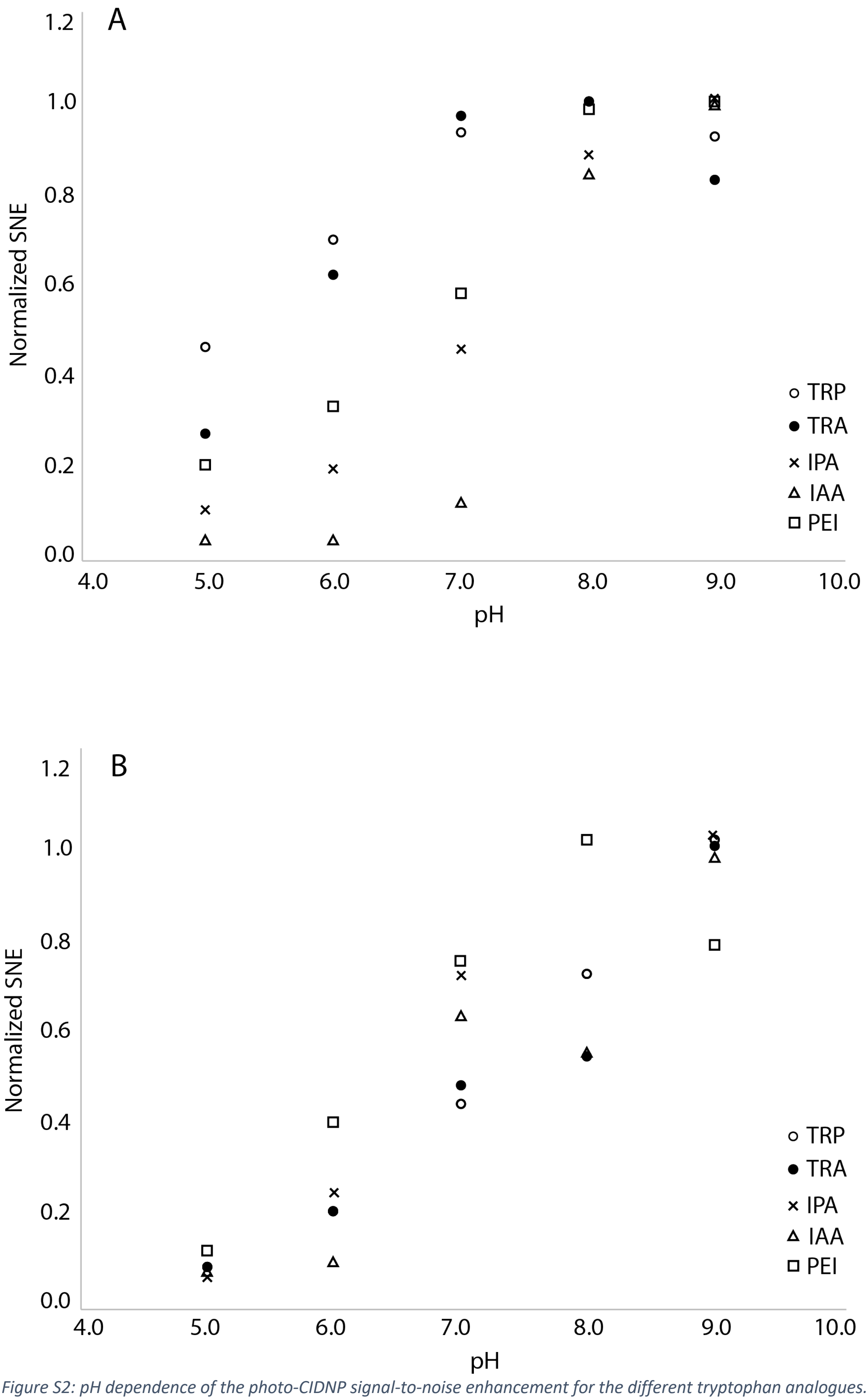

Figure S2: pH dependence of the photo-CIDNP signal-to-noise enhancement for the different tryptophan analogues. A) photo-CIDNP monitored by fluorescein. B) photo-CIDNP monitored by AT12. Because the enzyme cocktail used in other

measurements to prevent dye quenching is pH sensitive oxygen scavenging was performed using a cycle of vacuum and nitrogen atmosphere flush for 30 min, that yielded however in an overall less favorable SNE.

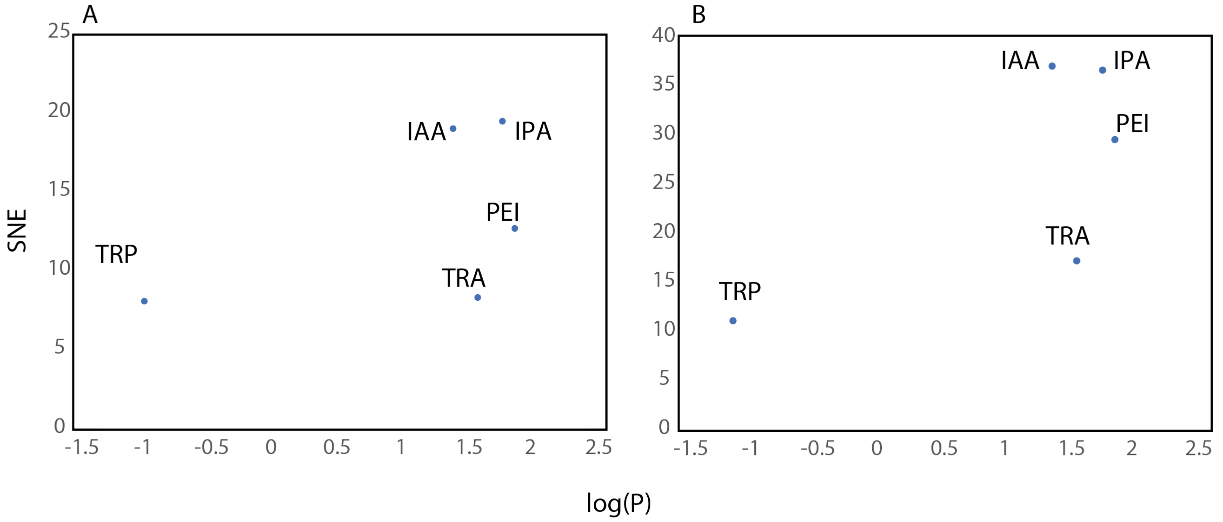

Figure S3: Signal-to-noise enhancements (SNE) for the different tryptophan analogues at higher pH. A) AT12, pH = 9. B) fluorescein pH = 8. Because the enzyme cocktail used in other measurements to prevent dye quenching is pH sensitive oxygen scavenging was performed using a cycle of vacuum and nitrogen atmosphere flush for 30 min, that yielded however in an overall less favorable SNE. Data on the dH-TRP is missing due to lack of sufficient available sample.

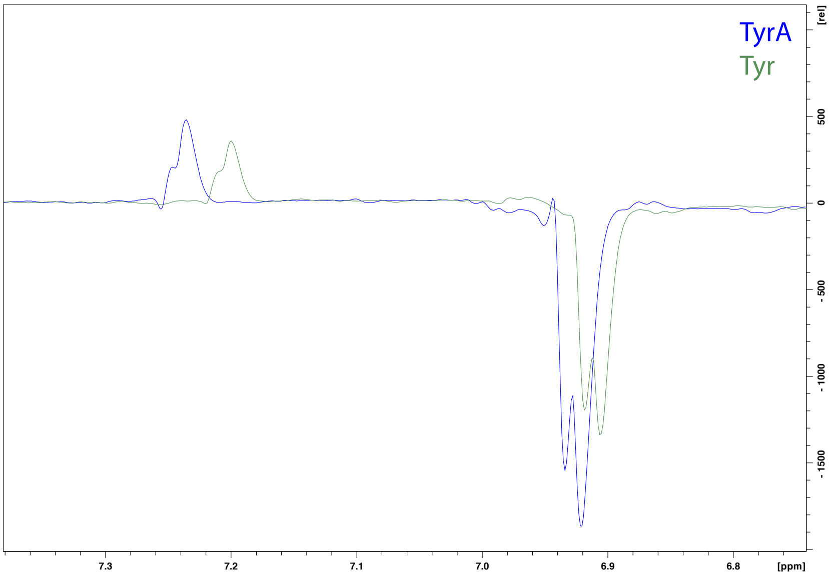

Figure S4: Photo-CIDNP spectra of tyrosine (Tyr) and tyramine (TyrA), with 45° detection pulses. Zoom on the aromatics. The samples were concentrated at 100 μM of Tyr/TyrA and 25 μM of AT12.

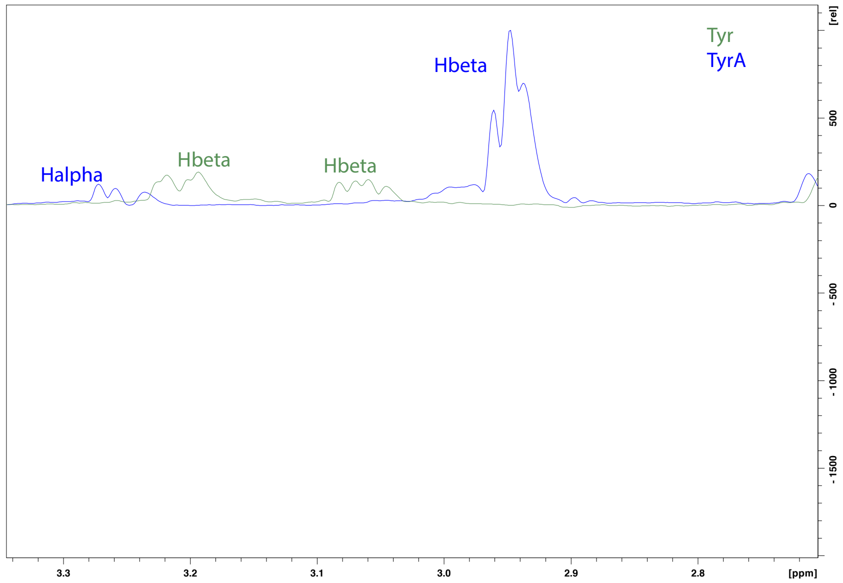

Figure S5: Photo-CIDNP spectra of tyrosine (Tyr) and tyramine (TyrA), with 45° detection pulses. Zoom on the aliphatics. The samples were concentrated at 100 μM of Tyr/TyrA and 25 μM of AT12.

Literature

Hore, P. J., and Broadhurst, R. W.: Photo-Cidnp of Biopolymers, Prog Nucl Mag Res Sp, 25, 345-402, 1993.
Kaptein, R., Nicolay, K., and Dijkstra, K.: Photo-Cidnp in Nucleic-Acid Bases and Nucleotides, Journal of the Chemical Society-Chemical Communications, 1092-1094, DOI 10.1039/c39790001092, 1979.
Okuno, Y., and Cavagnero, S.: Fluorescein: A Photo-CIDNP Sensitizer Enabling Hypersensitive NMR Data Collection in Liquids at Low Micromolar Concentration, Journal of Physical Chemistry B, 120, 715-723, 10.1021/acs.jpcb.5b12339, 2016.
Saprygina, N. N., Morozova, O. B., Grampp, G., and Yurkovskaya, A. V.: Effect of amino group charge on the photooxidation kinetics of aromatic amino acids, J Phys Chem A, 118, 339-349, 10.1021/jp4097919, 2014.
Sobol, A., Torres, F., Aicher, A., Renn, A., and Riek, R.: Atto Thio 12 as a promising dye for photo-CIDNP, J Chem Phys, 151, 234201, 10.1063/1.5128575, 2019.
Stob, S., and Kaptein, R.: Photo-Cidnp of the Amino-Acids, Photochem Photobiol, 49, 565-577, DOI 10.1111/j.1751-1097.1989.tb08425.x, 1989.
Tsentalovich, Y. P., Morozova, O. B., Yurkovskaya, A. V., Hore, P. J., and Sagdeev, R. Z.: Time-resolved CIDNP and laser flash photolysis study of the photoreactions of N-acetyl histidine with 2,2 '-dipyridyl in aqueous solution, Journal of Physical Chemistry A, 104, 6912-6916, 10.1021/jp000019o, 2000.
